# Effects of suprapubic photobiostimulation on bladder function in two girls with Hinman syndrome

**DOI:** 10.1007/s10103-026-04985-3

**Published:** 2026-08-12

**Authors:** Maria Verônica Pires, Jorge Nilton Casotti, Henrique Cunha Carvalho, Carlos José de Lima

**Affiliations:** 1 Biomedical Engineering Institute, Anhembi Morumbi University, São Paulo, Brazil; 2 Center for Innovation, Technology, and Education, São José dos Campos, Brazil; 3 Associação Distal Nefrologia e Urologia, Jacareí, Brazil; 4 Electronic Engineering Department, Federal University of Technology – Paraná, Campo Mourão, Brazil

**Keywords:** Dysfunctional voiding, Hinman syndrome, Photobiostimulation, Urodynamics

## Abstract

Hinman syndrome (HS) represents the severe clinical phenotype of dysfunctional voiding, characterized by functional bladder outlet obstruction in neurologically normal children. This study evaluated the effects of photobiostimulation (PBST) in two pediatric patients using serial urodynamic assessments. Two pediatric patients diagnosed with HS underwent a PBST therapeutic protocol based on light-emitting diodes (LEDs) emitting in the red spectrum (λ = 630 nm). Irradiation was applied over the suprapubic region. Baseline invasive urodynamic assessment (filling cystometry and pressure-flow study) was followed by serial free uroflowmetry during treatment to evaluate bladder emptying, voiding efficiency (VE), and detrusor kinetic parameters. At baseline, both patients exhibited detrusor hypocontractility and reduced emptying efficiency. Following PBST treatment, a reduction in post-void residual (PVR) of 93.6% was observed in patient 1 and 59.6% in patient 2, reflecting gains in VE of 277.4% and 18.7%, respectively. These preliminary findings suggest that PBST may improve bladder emptying in children with HS, as evidenced by increased voiding efficiency and improvements in detrusor kinetic parameters. The functional improvements are biologically plausible considering the known bioenergetic effects of PBST. The present findings support further investigation of suprapubic PBST as a non-invasive adjunctive therapy for pediatric dysfunctional voiding.

**Supplementary Information:**

The online version contains supplementary material available at 10.1007/s10103-026-04985-3.

## Introduction

Bladder function comprises low-pressure urine storage followed by efficient emptying, which depends on coordinated detrusor contraction with synchronous relaxation of the urethral sphincter and pelvic floor [1, 2]. This process is regulated by central and peripheral neural mechanisms, autonomic sensory afferents, neurotransmitters, and the structural integrity of the lower urinary tract [2]. Failure of coordinated relaxation of the urethral sphincter and pelvic floor during voiding may result in functional bladder outlet obstruction (BOO), progressive bladder remodeling, impaired detrusor contractility, and ultimately upper urinary tract deterioration [3–5].

Dysfunctional voiding (DV) is a common lower urinary tract dysfunction in children, ranging from mild functional alterations to complex conditions affecting the upper urinary tract. The most severe clinical phenotype, historically described as Hinman syndrome (HS), is characterized by functional bladder outlet obstruction in neurologically normal children, resulting in vesicosphincter discoordination, progressive bladder dysfunction, and potential upper urinary tract deterioration [3, 5, 6].

The therapeutic management of DV remains challenging, and no consensus exists regarding the optimal treatment strategy. Conservative treatment, including bladder retraining, pharmacotherapy, and biofeedback, constitutes the first-line approach [7, 8]. Patients refractory to conservative management may require botulinum toxin injection, sacral neuromodulation, or reconstructive surgery [3, 4, 9]. Therefore, less invasive therapeutic alternatives that can improve bladder function are needed.

Photobiostimulation (PBST), also referred to as photobiomodulation (PBM), is a non-invasive therapeutic approach based on the application of non-ionizing red (600–680 nm) or near-infrared (700–1000 nm) light. Its biological effects are primarily mediated by photon absorption by cytochrome c oxidase in the mitochondrial respiratory chain, resulting in increased ATP production and modulation of cellular pathways involved in tissue repair, inflammation, and neuromuscular function. These mechanisms may improve tissue bioenergetics and neural regulation, providing a biological rationale for investigating PBST as a potential treatment for lower urinary tract dysfunction [10, 11].

Experimental evidence suggests that PBST may also exert neuromodulatory effects, providing a potential mechanism for improving autonomic regulation of bladder function [12, 13]. Light-emitting diodes (LEDs) have become widely used in PBST because they allow safe, low-cost irradiation over larger tissue areas [14].

Based on the cited PBM mechanisms and the need for less invasive, more accessible therapies for children with HS, it was hypothesized that this approach could improve key parameters of bladder function. Therefore, the present study aimed to evaluate the effects of suprapubic PBST with 630 nm LEDs on bladder function in two pediatric patients diagnosed with HS through baseline urodynamic assessment and serial free uroflowmetry.

## Materials and methods

### Ethics and study design

The study was approved by the Research Ethics Committee of Anhembi Morumbi University (C.A.A.E No.: 88080525.2.0000.5492) and prospectively registered (11/04/2025) in the Brazilian Registry of Clinical Trials (ReBEC; RBR-3g8vcjs). All adults responsible for the participants signed a Free and Informed Consent Term (FICT). The study was conducted in accordance with the principles of the Declaration of Helsinki and the ethical standards established by the Brazilian National Health Council (Resolution No. 466/12).

A prospective exploratory case series was conducted in two neurologically normal girls, aged seven (Patient 1) and five years (Patient 2), diagnosed with Hinman syndrome. The duration of symptoms was three years for Patient 1 and one year for Patient 2. The diagnosis was established through comprehensive clinical evaluation, renal ultrasonography, renal scintigraphy (DMSA and DTPA), and standardized urodynamic assessment [5, 15].

Both patients had a history of recurrent urinary tract infections associated with impaired bladder emptying. Dynamic renal scintigraphy (DTPA) demonstrated delayed bladder emptying, which was subsequently confirmed by urodynamic assessment. Clinical manifestations included urgency, urinary incontinence, nocturia, and chronic constipation. Neurological and anatomical assessments excluded neurogenic bladder and anatomical bladder outlet obstruction. Neither patient was receiving clean intermittent catheterization or had undergone invasive treatment before enrollment. Patient 1 was subsequently diagnosed with attention-deficit/hyperactivity disorder (ADHD) after initiation of PBST; therefore, this diagnosis did not influence patient selection or the therapeutic protocol. Baseline assessment included clinical evaluation, free uroflowmetry (FU), and invasive urodynamic studies consisting of filling cystometry followed by pressure-flow studies (PFS) [5, 15, 16]. Invasive urodynamic assessment was performed only at admission to establish the diagnosis and characterize bladder dysfunction. During treatment, FU was repeated after completion of each PBST cycle to monitor changes in bladder emptying and voiding dynamics. After completion of the treatment protocol, both patients remained under monthly clinical follow-up for six months, including laboratory evaluation and free uroflowmetry.

### Urodynamic procedure

To assess bladder emptying and detrusor function post-void residual (PVR), voiding efficiency (VE), and voiding Time (T_M_ - total urinary flow duration) were analyzed because they provide clinically relevant information on voiding performance and bladder emptying under physiological conditions [5, 17, 18]. Classical Griffiths models [19, 20], based on the geometric relationship between detrusor contractile force, bladder geometry, and fiber shortening velocity, were applied to derive mean detrusor fiber shortening velocity (V_DETMED_) and detrusor fiber shortening velocity at maximum flow (V_DETQMAX_). These parameters enabled an integrated interpretation of pressure, urinary flow, and detrusor contractile kinetics throughout voiding.

According to the terminology of the International Children’s Continence Society (ICCS), detrusor underactivity was assessed during the voiding phase (pressure-flow study, PFS). In the present study, detrusor pressure (P_DET_) < 30 cmH₂O and P_DETQMAX_ <20 cmH₂O were adopted as criteria for detrusor hypocontractility based on previous pediatric urodynamic studies [5, 17, 19]. During the filling phase (cystometry), involuntary detrusor contractions (detrusor overactivity) are indicative of abnormal bladder storage function and, when associated with elevated storage pressures and reduced bladder compliance, increase the risk of vesicoureteral reflux, bladder trabeculation, and upper urinary tract damage [5, 16].

Free uroflowmetry (FU) was used as a physiological, non-invasive assessment of voiding that avoids potential interference associated with urethral catheterization [18]. Uroflowmetry curves were obtained when the child reported the desire to void, ensuring that voiding occurred close to the estimated optimal bladder capacity (OBC). Only recordings with a voided volume > 50% of the estimated OBC and > 50 mL were considered adequate. Recordings obtained with bladder filling < 50% or > 115% of the estimated OBC were excluded because they could distort detrusor mechanics and compromise the interpretation of voiding parameters [5, 16, 17].

### Equipment

The urodynamic study was performed using a urodynamic system (Dynapack MPX 816, Dynamed Ltda, Brazil) integrated with urodynamic analysis software (Urofive Plus, Dynamed Ltda, Brazil). A 6-Fr (about 2.0 mm diameter) urethral catheter was used to minimize artificial obstructive interference [20]. The voided volume was measured using an analytical balance (SF-400, Hardline, China). The PVR was determined immediately after voiding by ultrasonography using an ultrasound system (SonoAce 8000 EX, Samsung Medison Co., South Korea) equipped with a convex array probe transducer operating at a frequency of 3.5–5.0 MHz (Samsung Medison Co., South Korea).

### Bladder and voiding equations

The human bladder, when maximally filled at the threshold of voiding, tends to assume an approximately spherical geometry [21]. Accordingly, the equations used to calculate urodynamic and voiding parameters, including bladder volume (V), diameter (d), T_M_, P_DET_, V_DETMED_, voiding cycle, peak flow rate (Q_MAX_), PVR and VE, were based on previously published urodynamics studies [18, 20–23].

In this study, adequate VE was defined as ≥ 95% [22]. Comparing VE values before and after PBST, expressed as a percentage change, allowed quantification of the functional gain associated with the intervention.

### PBST device, dose and beam parameters

The initial therapeutic protocol consisted of three consecutive five-week PBST cycles, each including two treatment sessions per week (10 sessions per cycle). According to the study protocol, patients who did not achieve predefined functional treatment goals or showed inconsistent functional recovery after completion of the initial protocol were eligible for an additional three-cycle PBST treatment. A small, rigid, cylindrical device was constructed from polyvinyl chloride (PVC). The device comprises a circular polymeric flange with red LEDs (630 nm) arranged concentrically (Figure S1). The optical PBST dose and beam parameters are shown in Table S1, following the PBST report proposed by Jenkins and Carroll [24].

### Data analysis

The therapeutic response was evaluated by the individual percentage change (Δ%) between baseline and post-treatment values for each patient, using admission as the reference. Changes in PVR, VE, and detrusor kinetic parameters were analyzed descriptively. Data were analyzed using IBM SPSS Statistics (version 24.0; IBM Corp., Armonk, NY, USA) and OriginPro (version 8.5; OriginLab Corp., Northampton, MA, USA).

## Results

### Baseline urodynamic findings

Baseline urodynamic evaluation demonstrated increased bladder capacity, detrusor underactivity, increased PVR volume, and reduced VE in both patients, with more severe dysfunction in Patient 1. During filling cystometry, Patient 1 exhibited involuntary detrusor contractions consistent with non-incontinent detrusor overactivity. Baseline urodynamic findings are summarized in Tables 1 and 2.

### Bladder volume parameters in PBST treatment

**Fig. 1:**
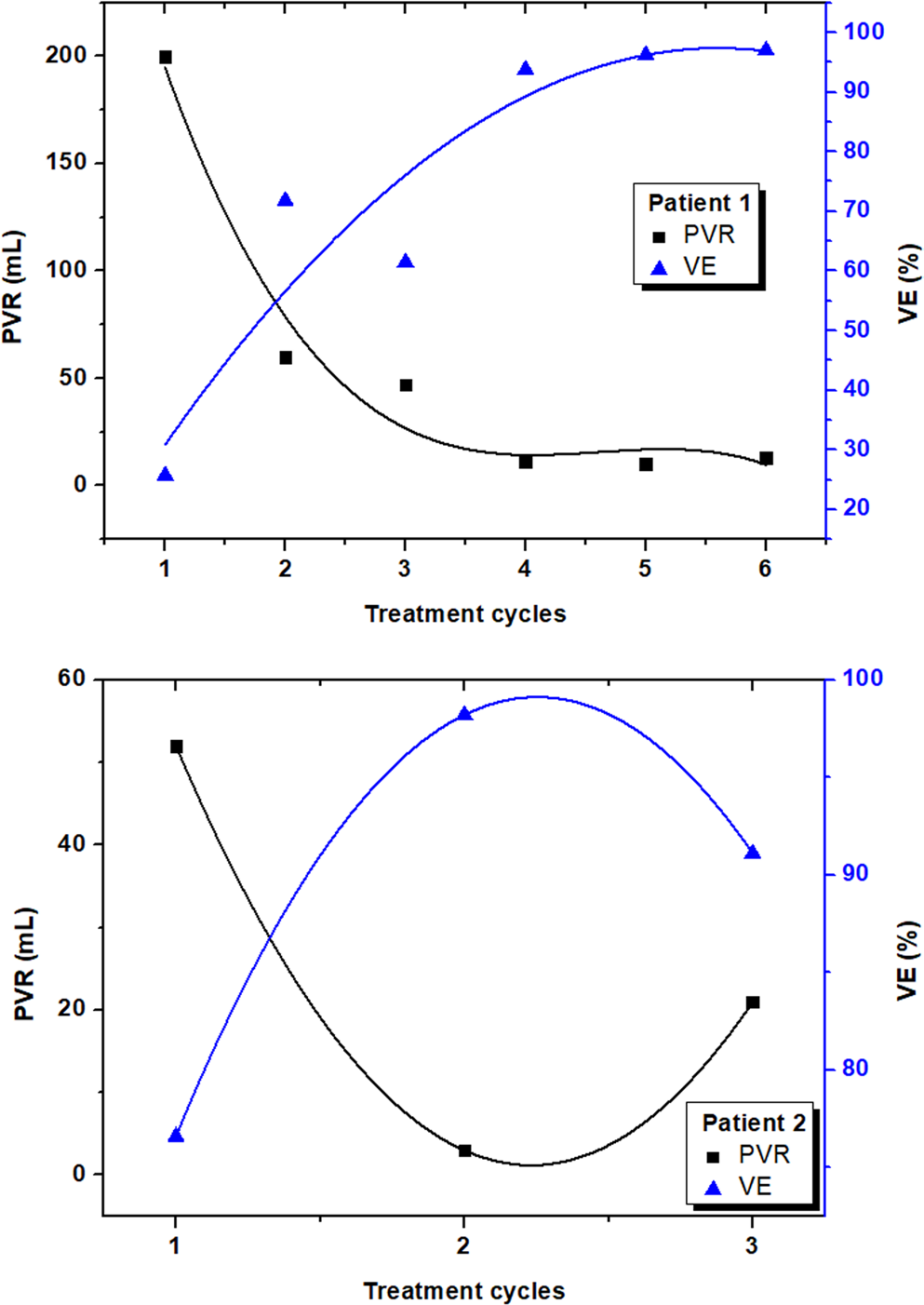
PVR and VE values for Patient 1 and 2 throughout PBST treatment cycles

Following PBST, both patients showed improvement in bladder emptying, characterized by marked reductions in PVR volume and corresponding increases in VE. After the initial treatment protocol, Patient 1 demonstrated a 76.5% reduction in PVR and a 139.3% increase in VE. Following the additional treatment protocol, cumulative improvement reached a 93.6% reduction in PVR, while VE increased to 97%, corresponding to an overall gain of 277.4% compared with baseline. Patient 2 also showed clinically meaningful improvement after the initial protocol, with a 59.6% reduction in PVR and an 18.8% increase in VE. Changes in bladder capacity and voided volume varied between patients and did not parallel improvements in bladder emptying. Detailed bladder volume parameters are presented in Table 1, and longitudinal changes in PVR and VE are shown in Fig. 1.

**Table 1:** Bladder volumes pre and post PBST treatment and percentage response per patient

Parameter	Patient 1						Patient 2		
	Pre	3rd	Δ_1_%	6th	Δ_2_%	Pre		3rd	Δ_1_%
Bladder Capacity (BC) (mL)	269	122	-54.6	399	48.3	223		238	6.72
Volume Drained (mL)	69	75	8.7	386	459.4	171		217	26.9
Post-Void Residual (PVR) (mL)	200	47	-76.5	12.8	-93.6	52		21	-59.6
Voiding Efficiency (VE) rate (%)	25.7	61.5	139.3	97	277.4	76.7		91.1	18.77

Patient 1: Post corresponds to the assessment after the additional treatment protocol. Patient 2: Post corresponds to the assessment after the initial treatment protocol

### Urodynamic and kinetic parameters

PBST was associated with improvements in urodynamic and detrusor kinetic parameters, particularly in Patient 1. Compared with baseline, maximum (Q_MAX_) and mean (Q_MED_) urinary flow rates increased by 966.6% and 1500%, respectively, while mean (V_DETMED_) and maximum (V_DETQMAX_) detrusor fiber shortening velocities increased by 2481% and 721%. These changes were accompanied by a 68.9% reduction in voiding time (T_M_), indicating substantial recovery of detrusor contractile performance. In contrast, Patient 2 exhibited only modest changes in flow and kinetic parameters despite clinically meaningful improvements in bladder emptying. Detailed urodynamic and kinetic results are presented in Table 2.

**Table 2:** Urodynamic and kinetic parameters of the detrusor obtained by FU in pre and post treatment periods

Parameter	Patient 1						Patient 2		
	Pre	3rd	Δ_1_%	6th	Δ_1_%	Pre		3rd	Δ_1_%
Q_MAX_ (mL/s)	3	2	-33.3	32	966.6	10		9	-10
Q_MED_ (mL/s)	1	1	-	16	1500	7		6	-14.29
T_M_ (s)	70.6	18.6	-73.6	21.9	-68.9	19.3		24.5	26.9
T_QMAX_ (mL/s)	9	18	-100	8.0	-11.1	7		12	71.43
V_DETMED_ (cm/s)	0.011	0.09	718.18	0.284	2481	0.149		0.173	16.11
V_DETQMAX_ (cm/s)	0.095	0.111	16.84	0.780	721	0.364		0.313	-14.01

Maximum flow (Q_MAX_ – mL/s), Mean flow (Q_MED_ - mL/s), Urinary flow duration (T_M_ - s), Time to maximum flow (T_QMAX_ - mL/s), Detrusor fiber shortening speed for maximum flow (V_DETQMAX_ - cm/s) and Mean detrusor fiber shortening speed (V_DMED_ - cm/s). Percentage difference between periods. Patient 1: Post corresponds to the assessment after the additional treatment protocol. Patient 2: Post corresponds to the assessment after the initial treatment protocol

## Discussion

The present study demonstrated that suprapubic PBST was associated with clinically meaningful improvement in bladder emptying in two children with HS. The most relevant findings were marked reductions in PVR, an increase in VE, and an improved in detrusor kinetic parameters, suggesting recovery of detrusor function after treatment.

Interpretation of these findings requires consideration of the baseline urodynamic abnormalities. Baseline urodynamic findings were consistent with the characteristic functional phenotype of HS, as evidenced by increased maximum cystometric capacity (MCC), elevated post-void residual volume, reduced voiding efficiency, and detrusor underactivity. MCC exceeded the estimated optimal bladder capacity by 50% in Patient 1 and 23.9% in Patient 2, findings consistent with prolonged functional bladder outlet obstruction and chronic detrusor dysfunction [3, 25]. Notably, although both patients presented enlarged bladder capacity at baseline, subsequent improvements in bladder emptying were not directly related to changes in this parameter. Patient 1 demonstrated a marked reduction, whereas Patient 2 showed a slight increase after treatment. These findings indicate that restoration of bladder emptying was more closely associated with improved detrusor performance than with changes in bladder distension.

The magnitude of the therapeutic response differed according to the baseline severity of bladder dysfunction. Patient 2 achieved satisfactory bladder emptying after the initial treatment protocol, whereas Patient 1 underwent an additional treatment protocol, resulting in a cumulative 93.6% reduction in post-void residual volume and restoration of voiding efficiency to 97%, indicating near-complete recovery of bladder emptying. These findings suggest that patients with more severe detrusor dysfunction may require longer PBST protocols to achieve optimal functional recovery. Although the mechanisms underlying these differences remain speculative, the observed functional improvements may be related to neuromodulatory effects of PBST on autonomic pathways involved in coordinated pelvic floor relaxation and detrusor activation during voiding.

The detrusor kinetic findings further support this interpretation. Among all evaluated urodynamic and kinetic parameters, mean detrusor fiber shortening velocity (V_DETMED_) emerged as the most sensitive functional marker of treatment response, closely paralleling improvements in bladder emptying. V_DETMED_, a parameter recently proposed based on Griffiths’ biomechanical principles [22], reflects the ability of the detrusor muscle to sustain contraction throughout voiding and therefore provides a physiological estimate of sustained detrusor contractile performance during bladder emptying. Patient 1 demonstrated a substantial increase in V_DETMED_ (+ 2481% compared with baseline), consistent with marked recovery of detrusor contractile performance, whereas Patient 2 exhibited only a modest improvement (+ 16.1%). These findings suggest that patients with more severe detrusor dysfunction may derive greater benefit from prolonged PBST treatment, although this observation requires confirmation in larger prospective studies.

The biological mechanisms underlying these functional improvements remain speculative. Experimental studies have shown that chronic bladder outlet obstruction impairs detrusor mitochondrial function, reducing oxidative phosphorylation and ATP production, thereby compromising sustained smooth muscle contraction and bladder emptying [25–28]. PBST has been shown to activate cytochrome c oxidase, enhancing mitochondrial bioenergetics, ATP synthesis, and intracellular calcium signaling. These bioenergetic and neuromodulatory effects may provide a plausible biological explanation for the improvements in detrusor function and bladder emptying observed in the present study [10, 11, 29].

A further consideration supporting the suprapubic application of PBST is that its biological effects are not exclusively dependent on direct photon penetration into the target tissue. Experimental and optical studies indicate that PBM can induce the release of photoactive mediators, including nitric oxide (NO), ATP, and other paracrine signaling molecules capable of propagating biological responses beyond the irradiated tissue. Accordingly, the functional improvements observed in the present study may have resulted from indirect neuromodulatory mechanisms rather than direct irradiation of the detrusor muscle. This hypothesis is consistent with the improvements in detrusor function and bladder emptying observed despite the limited direct tissue penetration expected for red light [14, 29–31].

Beyond the physiological findings, our results should also be interpreted within the broader biopsychosocial framework proposed for pediatric lower urinary tract dysfunction. Consistent with the recent review by Dawson et al. [32]., optimal management of these patients requires a multidisciplinary approach addressing behavioral, psychological, and functional factors. Although psychological outcomes were not evaluated in the present study, the objective improvements in bladder function observed after treatment suggest that PBST may serve as a useful adjunct to multidisciplinary management strategies for children with HS.

This study has several limitations. First, the rarity of HS and the inclusion of only two patients limit the generalizability of the findings and preclude statistical inference. Second, the absence of a control group prevents definitive conclusions regarding causality. In addition, individual differences in symptom duration and baseline severity of bladder dysfunction may have influenced the therapeutic response. Despite these limitations, both patients demonstrated consistent improvements across independent functional and urodynamic outcomes, including reductions in post-void residual volume, increased voiding efficiency, and improvements in detrusor kinetic parameters. These preliminary findings support the biological plausibility of suprapubic PBST and its potential as a non-invasive adjunctive therapy for children with HS, justifying further investigation in larger, controlled prospective studies.

## Conclusion

Suprapubic PBST using 630-nm LEDs was associated with clinically meaningful improvement in bladder emptying in two children with dysfunctional voiding presenting the severe clinical phenotype historically described as HS. Treatment resulted in marked reductions in PVR volume, increased VE, and improvements in detrusor kinetic parameters, supporting recovery of detrusor function. The cumulative improvement observed after an additional treatment protocol in the patient with more severe baseline dysfunction suggests that therapeutic response may depend on disease severity and treatment duration. Although limited by the small sample size, these findings provide preliminary evidence supporting the biological plausibility of suprapubic PBST as a non-invasive adjunctive therapy for children with dysfunctional voiding.

## Supplementary information

Below is the link to the electronic supplementary material.

Supplementary Material 1

## Data Availability

No datasets were generated or analysed during the current study.
