# Supplementary material for: Effects of suprapubic photobiostimulation on bladder function in two girls with Hinman syndrome
Source: Lasers Med Sci. 2026 Aug 12;41(1):187. doi: 10.1007/s10103-026-04985-3 (PMC13468988; doi:10.1007/s10103-026-04985-3)
Supplement: Supplementary file 1 [file 10103_2026_4985_MOESM1_ESM.docx]

**Supplementary Material**

Table S1. Photobiomodulation dose and beam parameters [24].

Device information.

| Manufacturer | Own development and assembly |
| --- | --- |
| Model Identifier | NA |
| Number of Emitters | 35 |
| Emitter Type | GaAlAs LED (5 mm diameter encapsulation) |
| Spatial Distribution of Emitters | LEDs arranged geometrically in concentric circles |
| Beam Delivery System | hand-held probe to suprapubic region |

NA: Not Applicable.

Irradiation parameters.

| Parameter [unit] | Value | Measurement method or information source |
| --- | --- | --- |
| Center wavelength [nm] | 630 | Manufacturer’s datasheet |
| Spectral bandwidth [nm] | 22 | Manufacturer’s datasheet |
| Operating mode | Continuous wave (CW) | - |
| Frequency [Hz] | NA | - |
| Pulse on duration [sec] | NA | - |
| Pulse off duration [sec] or duty cycle [%] | NA | - |
| Energy per pulse [J] | NA | - |
| Peak radiant power [mW] | NA | - |
| Average radiant power [mW] | 17 (each LED emitter) | Optical Power Meter, PM100D, Thorlabs Corp., NJ, USA |
| Polarization | NA | - |
| Aperture diameter [cm] | 5 | - |
| Irradiance at aperture [mW/cm^2^] | 30 | Author calculated |
| Beam divergence [deg] | 20 | Manufacturer’s datasheet |
| Beam shape | Circular | Manufacturer’s datasheet |
| Beam profile | Top hat | Manufacturer’s datasheet |

Treatment parameters.

| Parameter (unit) | Value |
| --- | --- |
| Beam spot size at target [cm^2^] | 20 |
| Irradiance at target [mW/cm^2^] | 30 |
| Exposure duration [sec] | 333 |
| Radiant exposure [J/cm^2^] | 10 |
| Radiant energy [J] | 200 |
| Number of points irradiated | 1 |
| Area irradiated [cm^2^] | 20 |
| Application technique | Touching/contact with the suprapubic region |
| Number and frequency of treatment sessions | Twice a week for 5 weeks and after a 30-day interval, three times (30 sessions) |
| Total radiant energy [J] | 200 (per session) and 6.000 (over all sessions) |

Figure S1. Photobiomodulation (PBM) red-LED device.

Figure S2. Urodynamic tracing from Patient 1 and 2.
